# Forest gaps alter the soil bacterial community of weeping cypress plantations by modulating the understory plant diversity

**DOI:** 10.3389/fpls.2022.920905

**Published:** 2022-08-19

**Authors:** Qian Lyu, Yan Luo, Size Liu, Yan Zhang, Xiangjun Li, Guirong Hou, Gang Chen, Kuangji Zhao, Chuan Fan, Xianwei Li

**Affiliations:** ^1^College of Forestry, Sichuan Agricultural University, Chengdu, China; ^2^Sichuan Academy of Forestry, Chengdu, China; ^3^Key Laboratory of National Forestry and Prairie Bureau on Forest Resources Conservation and Ecological Security in the Upper Reaches of Yangtze River, Sichuan Agricultural University, Chengdu, China; ^4^Forestry Ecological Engineering in Upper Reaches of Yangtze River Key Laboratory of Sichuan Province, Sichuan Agricultural University, Chengdu, China

**Keywords:** forest gap sizes, understory vegetation, near-natural forest management, soil bacteria, soil properties

## Abstract

Weeping cypress is an endemic tree species that is widely planted in China, and the simple stand structure and fragile ecosystem of its plantation are common issues. Exploring the effect of different gap sizes on the soil bacterial community structure of weeping cypress plantations can provide a theoretical basis for the near-natural management of forest plantations. We, therefore, constructed three kinds of forest gaps with different sizes in weeping cypress plantations, namely, small (50–100 m^2^), medium (100–200 m^2^), and large gaps (400–667 m^2^), for identifying the key factors that affect soil bacterial communities following the construction of forest gaps. The results suggested that the herb layer was more sensitive than the shrub layer, while the Simpson, Shannon, and richness indices of the herb layer in plots with gaps were significantly higher than those of designated sampling plots without any gaps (CK). The presence of large gaps significantly increased the understory plant diversity and the Shannon and Simpson indices of the soil bacterial alpha diversity. There were obvious changes in the community composition of soil bacteria following the construction of forest gaps. The dominant bacterial phyla, orders, and functions were similar across the plots with different gap sizes. Of the indicator bacterial species, the abundance of the nitrogen-fixing bacteria, *Lysobacter_ yangpyeongensis*, and *Ensifer_meliloti*, was significantly different across plots with different gap sizes and accounted for a large proportion of the bacterial population of plots with medium and large gaps. The understory plant diversity was mostly related to the soil bacterial community than to other soil factors. The results of structural equation modeling indicated that the understory plant diversity was the most important environmental factor in driving the composition and diversity of bacterial communities. The construction of forest gaps significantly improved the understory plant diversity, physicochemical properties of the soil, and bacterial diversity in weeping cypress plantations, and the results of the comprehensive evaluation were in the order: large gaps > small gaps > medium gaps > CK. Our results suggested that large gaps are beneficial for the diversity of above-ground plant communities and underground soil bacterial communities.

## Introduction

Forests are under pressure for providing ecosystem services to societies worldwide, and there is an increasing necessity for protecting the biotic components of forests (Erasmy et al., 2021). Forests have a variety of ecological service functions, providing ecosystems with resistance to external interference and enhancing the resilience of disturbed forest ecosystems (Li W. et al., 2020; Li X. et al., 2020). Forest plantations are an important component of global forest resources, and their construction has greatly increased the global forest coverage and resolved the deficit between wood supply and demand. However, the challenges to forest management strategies result in the instability of plantation ecosystems (Payn et al., 2015). Therefore, achieving ecological benefits from plantation management is particularly crucial.

With the recent increase in effective forest management strategies, the artificial formation of forest gaps by harvesting trees is being considered to be a sustainable practice (Li et al., 2019). As a common disturbance-based forest management strategy, forest gaps play an essential role in forest ecology, affecting biological dynamics, nutrient cycling, and plant succession (Yang et al., 2017). The environmental heterogeneity of forest gaps plays a major role in increasing plant diversity (Swartz et al., 2020). The proportion of incident sunlight under the forest canopy and the exposed area of the woodland increases following the formation of forest gaps, which directly affect species composition and community structure of the understory vegetation (Hubbell et al., 1999; Tena et al., 2020). Some studies demonstrated that the herb layer is more sensitive to environmental changes than the tree and shrub layers and is more likely to reflect the effect of the transformation within a short period of time (Graf et al., 2019; Abbas et al., 2020). Plants in the herb layer are expected to serve as ecological indicators for monitoring the biodiversity and sustainability of forest management strategies (Von Oheimb and Härdtle, 2009). Therefore, based on the results of previous studies, we first hypothesized that forest gaps can increase the diversity of understory plants, and changes in the herb layer are more pronounced than those in the shrub layer.

Soil plays a pivotal role in the flow pattern of basic nutrients and energy in forest ecosystems (Adamo et al., 2021). Soil microorganisms, especially soil bacteria, primarily regulate the functions of soil ecosystems, including soil nutrient cycling and decomposition of organic matter (Liu J. et al., 2021; Liu Y. et al., 2021). Stand disturbance can affect the diversity and composition of soil bacterial communities. Numerous studies demonstrated that forest management strategies significantly affect the composition of soil bacterial communities; however, the composition of dominant bacterial phyla, classes, genera, and other species is similar (Fierer et al., 2012; Jin et al., 2019; Lyu et al., 2022). Bacterial alpha diversity promotes numerous soil processes (Delgado-Baquerizo et al., 2018), and a higher environmental heterogeneity increases the alpha diversity of organisms (Stein et al., 2014). Typically, plant and animal diversity increases significantly with an increase in environmental heterogeneity (Curd et al., 2018). In this study, we aimed to verify whether this pattern is observed in bacterial diversity as well. We hypothesized that an increase in the size of forest gaps might increase the alpha diversity of soil bacteria, while the composition of the dominant phyla, orders, and species would remain similar at different gap sizes. We further hypothesized that the structure of the soil bacterial community would undergo alterations following the construction of forest gaps.

Bacterial communities in forest soils are affected by numerous biotic and abiotic factors, including temperature, precipitation, above-ground plant distribution, forest management measures, and other edaphic properties (Jin et al., 2019; Zeng et al., 2019). Soil pH is the primary factor that affects the composition and diversity of soil bacterial communities (Ramirez et al., 2014; Liu et al., 2020). In addition, it has been demonstrated that soil pH has a greater effect on the composition of soil bacterial communities than vegetation types and plant diversity (Cheng et al., 2020). However, in view of the close relationship between plants and soil microorganisms, it is considered that plant diversity is related to the diversity and structure of soil bacteria, owing to the diversity of litter types and root exudates (Wardle et al., 2004; Haichar et al., 2008; Li et al., 2015). For instance, the production of litter, root exudates, and plant exudates is affected by abundant understory vegetation, which has well-developed root systems (Yang et al., 2015). The intertwining of root systems enhances the population and activity of soil microbes, which in turn affects the distribution of soil bacterial communities (Urbanová et al., 2015). Although the main factors affecting the composition and diversity of soil bacterial communities have been widely studied (Nacke et al., 2011; Liu et al., 2013; Jin et al., 2019), the driving factors affecting the community structure of soil bacteria due to the reconstruction of forest gaps remain to be elucidated. Therefore, considering that the first two assumptions are true, we hypothesized that the diversity of understory plants plays an important role in altering the community structure of soil bacteria.

To confirm the aforementioned three hypotheses, we selected the weeping cypress (*Cupressus funebris*) plantations of the Yangtze River shelterbelt project as the research object and adopted three different forest gap sizes. Weeping cypress plantations without opening gaps were considered as the control for (1) exploring the alterations in understory plant diversity at different gap sizes, (2) evaluating the alterations in the diversity and composition of the soil bacterial community at different gap sizes, (3) and determining whether understory plant diversity affects the community structure of soil bacteria.

## Materials and methods

### Study site

The study was conducted in Yufeng town (30° 25′ 06″ N, 105° 32′ 19″ E) in Suining County, Sichuan Province. The altitude ranged from 300 to 600 m. The area has a subtropical monsoon climate, which is characterized by a mild climate with four distinct seasons and a long frost-free period, an annual average temperature of 17.4°C, sufficient heat, and abundant rainfall, with an average annual rainfall of 930 mm. The study area has pure stands of cypress forests made by the long-term forest afforestation project of 1990, with a single-stand structure, low plant diversity, low resistance to diseases and insect pests, and in a state of degradation. The area has calcareous purple soil with poor fertility. The dominant understory species are *Coriaria nepalensis, Myrsine africana, Rhus chinensis, Vitex negundo, Ficus tikoua*, and *Stenoloma chusanum*.

### Experimental design and soil sampling

The sizes of the forest gaps were measured in October 2015 using a laser distance meter (LDM-80H). The experiment was a completely randomized design, and nine plots with artificial forest gaps and three control plots without forest gaps (CK), having similar elevation and slope, were selected in the permanent plot. The sizes of the artificially constructed forest gaps ranged from 50 to 667 m^2^. The gaps were additionally divided into three categories, namely, small gaps (S): 50–100 m^2^ (*n* = 3), medium gaps (M): 100–200 m^2^ (*n* = 3), and large gaps (L): 400–667 m^2^ (*n* = 3) (Zhu et al., 2015), surrounded by closed canopy transition zones and a 5-m buffer (Lyu et al., 2021a). All the gaps were approximately oval, with west-facing slopes ranging from 13° to 15°. The mean height of the gap border trees was 12.67 m, with a mean breast diameter of 10.96 cm. The trunks and branches of the weeping cypress trees that had been felled for the construction of forest gaps were removed, and plant regeneration in the gaps primarily depended on seeds and the soil seed bank.

Soil sample collection and vegetation surveys were conducted in October 2019. A soil core (10 cm in depth and 5 cm in diameter) was used for collecting soil samples from the top 10 cm of the soil profiles following the removal of litter horizons from each plot. Soil samples were collected from five sampling points in the four corners and center of the plots, and the five samples were mixed to obtain one composite sample. The evenly composite soil sample was divided into two samples. Each of the samples of fresh soil was packed in a plastic bag within 24 h to the laboratory in a portable container maintained at 4°C. Following the removal of visible roots, residues, and rocks, one of the samples was placed in a freezer at −80°C for microbial analysis, while the other was divided into two subsamples, one of the subsamples was stored in a refrigerator at 4°C for the determination of soil microbial biomass carbon (MBC) and microbial biomass nitrogen (MBN) of the soil, while the other subsample was air-dried and used to determine the physicochemical properties of the soil.

### Analyses of soil properties and understory plant diversity

Soil temperature (ST) and soil moisture (SM) were recorded by a Thermochron iButton Device (DS1921-G, Maxim Integrated, San Jose, CA, USA). Soil samples were weighed, oven-dried at 105°C, and weighed again. Soil bulk density (SBD) was obtained by dividing the dry soil mass by the (known) volume of the sampling core and expressed as g/cm3 (Mora and Lázaro, 2014). The content of soil organic matter (SOM) was determined by hydration with the potassium dichromate oxidation-colorimetric method. The content of soil hydrolyzable nitrogen (AN) was measured using the alkali-hydrolyzed diffusion absorption method (Chen et al., 2010). The total nitrogen (TN) content was determined using the Kjeldahl method (Stanford et al., 1973), while the content of soil-available phosphorus (AP) was measured by extracting the subsamples with 0.03 M NH_4_F−0.025 M HCl (Mariotte et al., 2020). The total phosphorus (TP) content was measured using the alkali fusion-Mo-Sb anti-spectrophotometric method (Ren et al., 2019). The content of MBC and MBN in the soil samples was estimated using the chloroform fumigation-extraction method with a total organic carbon (TOC) analyzer (LIQUIC TOCII, Elementar Analysensysteme GmbH, Hanau, Germany).

Five 3 × 3 m squares in the four corners and center of the sampling squares were mechanically designated as shrub quadrats in the gap areas of each of the weeping cypress plantations, following which ten 1 × 1 m squares were designated in each plot as herb quadrats. The species names, the number of plants, branch diameter, and height of each plant in the shrub quadrats were determined and recorded. The average height and branch diameter of each of the shrubs in the plots were measured using a tape line. The names of the species in the herb layer, their overall coverage, the average coverage, and the average height of each species in the herb quadrats were determined and recorded. The understory plant diversity was evaluated with four alpha-diversity indices, namely, richness (*R*), the Shannon–Wiener index (*H*), the Simpson index (*D*), and the Pielou index (*J*).

### Soil DNA extraction

Total DNA was isolated from 0.5 g of soil using HiPure Soil DNA kits (Magen, Guangzhou, China). The DNA concentration was determined using a NanoDrop spectrophotometer (NanoDrop2000, Thermo Scientific, Wilmington, DE, USA), and the DNA samples were stored in a freezer at −80°C for further analyses.

### PCR amplification and 16S sequencing

The genes in the V3-V4 variable regions of 16S were amplified using the specific 341F-806R primer pair. The products of polymerase chain reaction (PCR) were accumulated, and the products from the first round of PCR were subsequently purified using AMPure XP Beads and quantified using a Qubit 3.0 fluorometer. The products from the second round of amplification were purified using AMPure XP Beads. The amplicons extracted with 2% agarose gels were purified using AMPure XP Beads (Beckman Coulter, Inc., CA, USA) and subsequently quantified using an ABI StepOnePlus Real-Time PCR System (Life Technologies, Foster City, USA). The purified amplicons were pooled in equimolar quantities and paired-end sequenced (PE250) on an Illumina NovaSeq 6000 sequencing platform by Gene De novo Biotechnology Co., Ltd. (Guangzhou, China).

After the polymerase chain reaction products were gathered together, the first round of PCR products was purified, the PCR products were purified by AMPure XP Beads and then quantified by Qubit3.0, and the second round of amplification products was purified by AMPure XP Beads. The amplicons extracted from 2% agarose gels were purified using AMPure XP Beads (Beckman Coulter, Inc., CA, United States) and then quantified using an ABI StepOnePlus Real-Time PCR System (Life Technologies, Foster City, United States). Purified amplicons were pooled in equimolar amounts and paired-end sequenced (PE250) on an Illumina NovaSeq 6000 by Gene De novo Biotechnology Co., Ltd. (Guangzhou, China).

The raw reads were sequenced, and the low-quality reads were filtered to obtain effective data (clean data). The clean data were assembled and refiltered to ensure clustering of the most effective data into operational taxonomic units (OTUs). After the OTUs were obtained, the community functions were predicted according to the analyses performed, including species annotation, the alpha-diversity analysis, the beta-diversity analysis, and the PICRUSt analysis (Langille et al., 2013). The differences between groups were compared and tested in the case of effective grouping. Finally, the relationship between the microorganisms and the environment was determined from various factors, including environmental factors, combined with specific analytical methods, including principal coordinate analysis (PCoA), principal component analysis (PCA), and redundancy analysis (RDA). The species diversity information of the samples was studied by clustering all the effective tag sequences of all the samples using Uparse software v.9.2.64. By default, the sequences were clustered into OTUs at 97% consistency (identity), and the tag absolute abundance and relative information of each OTU in each sample were calculated. The archaea and unclassified bacteria were removed for reducing the influence of low-abundance OTUs on the results of the entire analysis, and OTUs with a total abundance of 1 were filtered and excluded in the follow-up analysis (Aßhauer et al., 2015). In this study, the function of the bacterial community was analyzed using the PICRUSt software. Six types of biological metabolic functions, namely, level 1, metabolism, genetic information processing, cellular processes, environmental information processing, and organismal systems and human diseases, were obtained by comparing with the KEGG database. The raw reads were deposited into the National Center for Biotechnology Information (NCBI) Sequence Read Archive (SRA) database (BioProject: PRJNA784027).

### Statistical analyses

PCoA and permutational multivariate analysis of variance (PERMANOVA) based on the Bray–Curtis distance were conducted in R 3.4 (http://www.r-project.org/) for evaluating the overall changes in the soil bacterial community structure across different gap sizes. One-way ANOVA was used for evaluating the physicochemical properties of the soil, undergrowth plant diversity, diversity and composition of the soil bacterial community, and composition of the soil bacterial functional groups in the plots with three different gap sizes. The labdsv package in R was used to determine the indicator value of the species with abundance value > 0 and total proportion > 0.1% in each sample of the comparison groups. Cross-validation was performed during statistical analyses and the determination of *p*-values. The indicator species were analyzed for identifying the potential biomarkers in the regions with three different gap sizes. Pearson's correlation analysis was used for evaluating the relationships between the indicator species in the soil bacterial community and environmental variables. Pearson's correlation analysis was performed for determining the relationship between the environmental variables and the alpha diversity of soil bacteria. Variance partitioning analysis (VPA) was performed for determining the explanation of each of the environmental factor variables for the total variation in the composition and distribution of soil bacterial communities at the phylum level. RDA of the physicochemical properties of the soil and understory plant diversity was performed using CANOCO 5.0. The function of the bacterial community was analyzed using PICRUSt. The principal component variables were extracted from the comprehensive indices of the physicochemical properties of the soil, plant diversity, and bacterial diversity by PCA, and a comprehensive evaluation value was calculated using a comprehensive evaluation model.

Structural equation modeling (SEM) was employed for exploring the effects of the physicochemical properties of the soil and understory plant diversity on the diversity (Simpson index) and composition (using phylum-level abundance data) of the soil bacterial community. Based on published studies on the relative contributions and interactions of variables (Jin et al., 2019), the analysis was performed with AMOS 26.0 software using robust maximum likelihood estimation procedures. Prior to SEM, a non-metric multidimensional scaling analysis was performed using the bacterial sequencing data at the phylum level and plant diversity indices of the herb and shrub layers. The respective first-axis data were subsequently selected and incorporated as the bacterial composition, shrub diversity, and herb diversity into the model constructed by SEM (Chen et al., 2020). Several tests were performed for assessing the fitness of the model, including chi-square tests and assessment of the obtained *p*-value, determination of the comparative fit index (CFI), goodness-of-fit (GFI), Akaike's information criterion (AIC), and the root square mean error of approximation (RMSEM). Based on the results of GFI tests, we excluded fewer predictive measures, nonsignificant indicators, and nonsignificant pathways and retained the most informative variable (Tu et al., 2022). The final models that best fitted our data were obtained by the stepwise removal of nonsignificant components from the model.

## Results

### Physicochemical properties of the soil and understory plant diversity

Compared to that of the CK plots, the TN content decreased significantly in the plots with small gaps but increased significantly in those with medium and large gaps (*p* < 0.05). There were significant differences in the SOM, AP, and SBD of plots with small forest gaps. There were significant differences between the TP content of CK plots and those with small and medium forest gaps (*p* < 0.05). The size of the forest gaps had no significant effect on the pH, AN, MBC, MBN, SM, and ST (*p* > 0.05; Supplementary Table S1).

Compared with those of the CK sampling plots, there were significant differences in the Simpson index of plots with medium gaps (*p* < 0.05) and the Shannon index of plots with large gaps in the shrub layer (*p* < 0.05), while no significant differences were observed in the Pielou index (*p* > 0.05). There was a significant difference between the richness index of plots with medium gaps and large gaps (*p* < 0.05). The Simpson, Shannon, and richness indices of the herb layer of plots with forest gaps were significantly higher than those of the CK plots (*p* < 0.05). The Pielou index of the sampling plots with medium and large gaps was higher than that of the CK plots, but there was no significant difference between the Pielou index of the plots with small gaps and the CK plots (*p* > 0.05; Table 1).

**Table 1:** Understory plant diversity indices of herbs and shrubs at different canopy gap levels.

	**Canopy gap levels**	**Simpson**	**Shannon-Wiener**	**Pielou**	**Richness**
Shrub	CK	0.90 ± 0.0095b	1.05 ± 0.0465b	0.93 ± 0.0037a	13.67 ± 1.5281c
	S	0.91 ± 0.0032ab	1.09 ± 0.0055b	0.95 ± 0.0481a	14.00 ± 0.0032c
	M	0.95 ± 0.0428a	1.14 ± 0.1215b	0.94 ± 0.0488a	21.00 ± 0.0027b
	L	0.93 ± 0.0014ab	1.27 ± 0.0047a	0.91 ± 0.0034a	25.00 ± 0.0047a
Herb	CK	0.71 ± 0.0024c	0.60 ± 0.0045c	0.85 ± 0.0026b	5.00 ± 0.0018d
	S	0.87 ± 0.0064b	0.96 ± 0.017a	0.87 ± 0.0182b	12.67 ± 0.5801a
	M	0.86 ± 0.0028b	0.90 ± 0.0039b	0.92 ± 0.0209a	9.67 ± 0.5771c
	L	0.88 ± 0.0020a	0.96 ± 0.0018a	0.92 ± 0.0017a	11.00 ± 0.0023b

*Different letters indicate significant differences (P < 0.05) among canopy gap levels, based on a one-way ANOVA followed by an LSD test. “S” stands for small gaps; “M” stands for medium gaps; and “L” stands for large gaps*.

**Figure 1:**
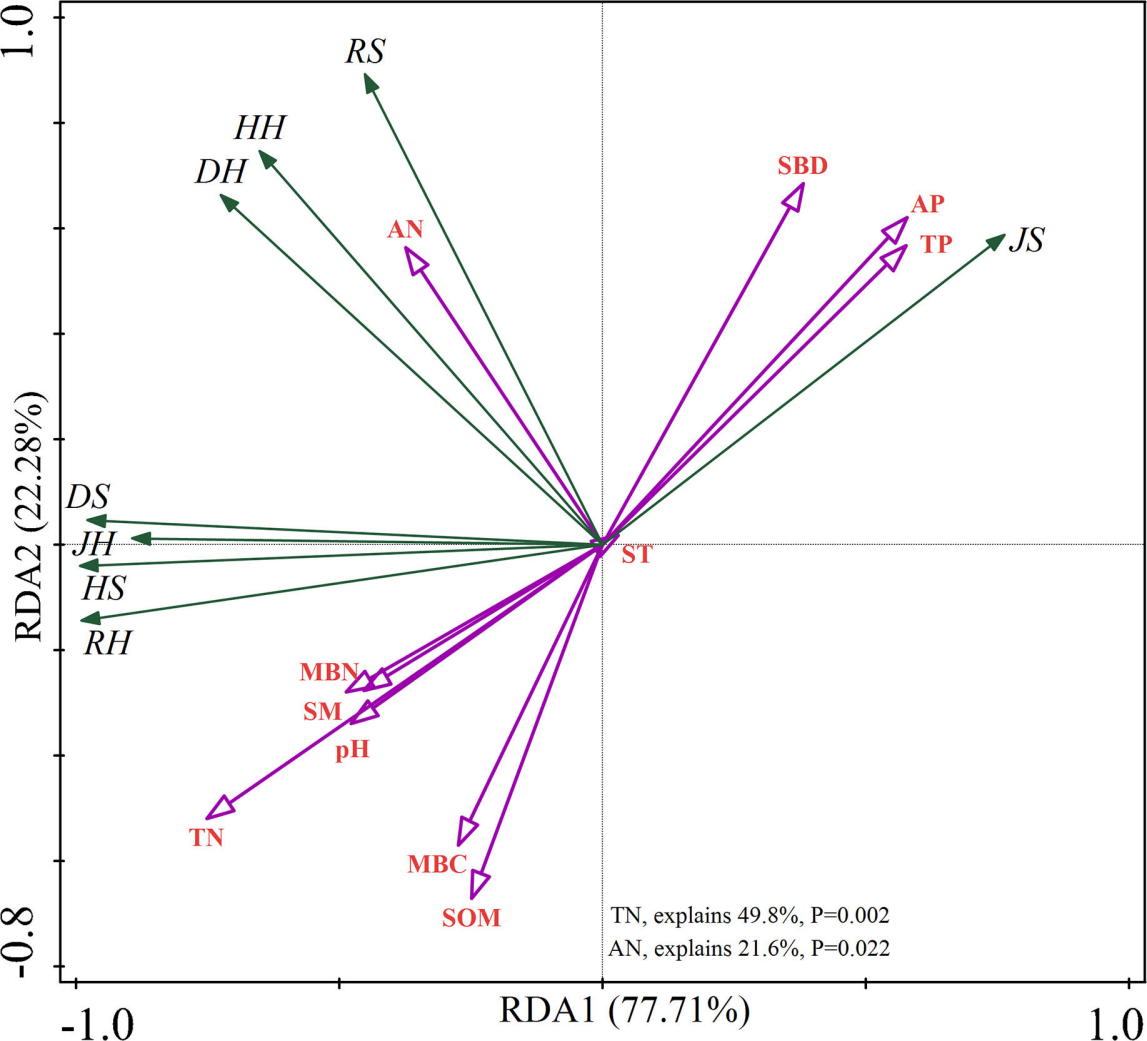
Redundancy analysis (RDA) of understory plant diversity with soil properties. “S” stands for small gaps; “M” stands for medium gaps; and “L” stands for large gaps.

The RDA showed that the total contribution of soil physicochemical properties to the understory plant diversity was 99.99% (Figure 1). TN and AN had a significant impact on understory plant diversity (*p* < 0.05), which explained 71.40% of the variation and contributed to understory plant diversity.

### Diversity and composition of the soil bacterial community

**Figure 2:**
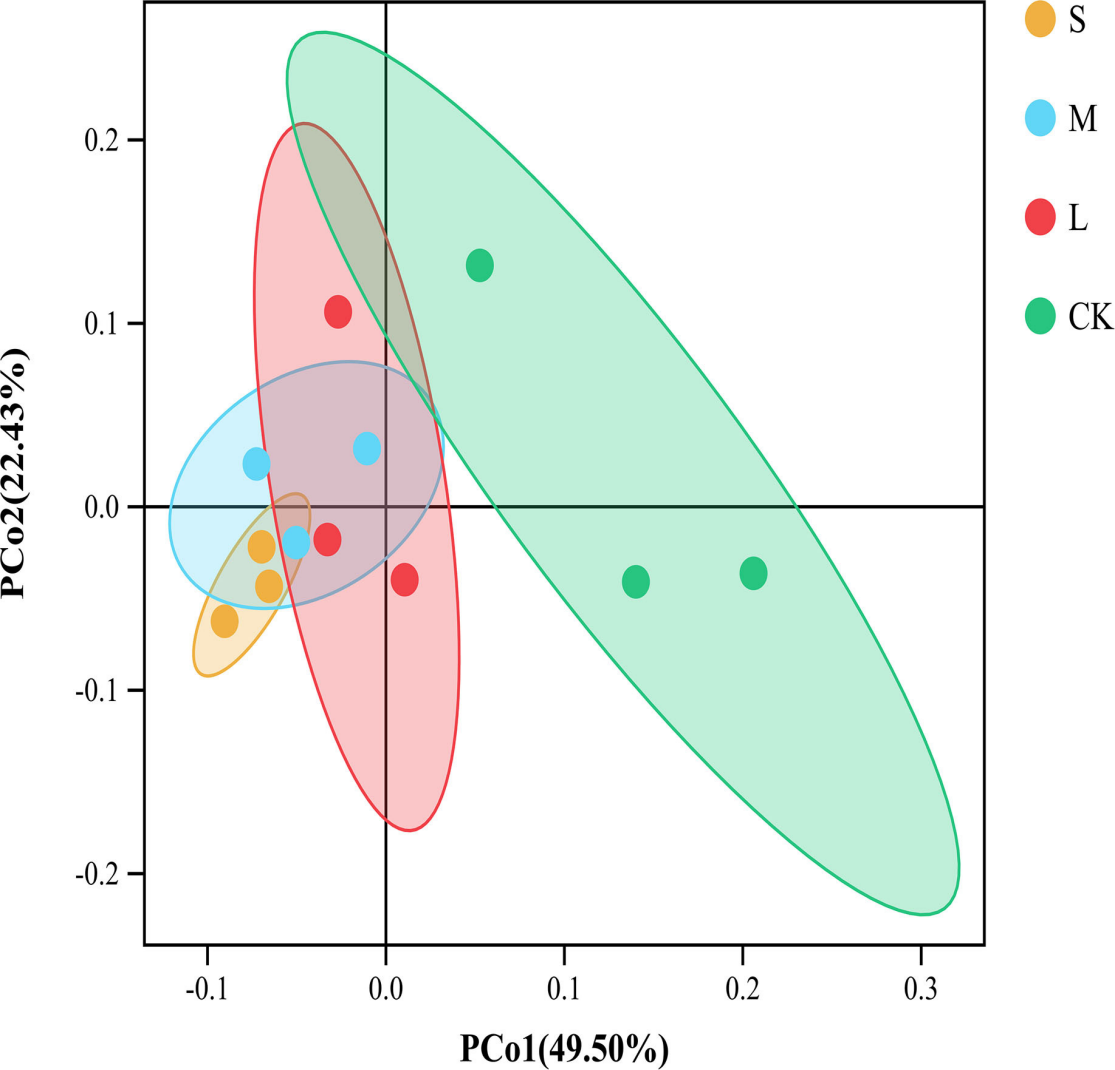
Principal coordinate analysis (PCoA) of the soil bacterial community based on Bray–Curtis distance among different groups. The ellipses represent the 95% confidence interval. “S” stands for small gaps; “M” stands for medium gaps; and “L” stands for large gaps.

We obtained a total of 934,853 bacterial effective tags from the raw dataset, which were clustered into 11417 OTUs. There was no significant difference between the Chao1 and ACE indices of the CK plots and the sampling plots with small, medium, and large gaps. The Shannon and Simpson indices of plots with large gaps were significantly higher than those of the CK plots (*p* < 0.05; Table 2). The results of PCoA and the analysis of similarities showed significant variations (R = 0.497, *p* = 0.002) in the soil bacterial community structures across the sampling plots with different gap sizes (Figure 2; Supplementary Figure S1).

**Table 2:** Bacterial diversity of different canopy gap levels.

**Canopy gap levels**	**Shannon**	**Simpson**	**Chao1**	**ACE**
CK	9.76 ± 0.15b	0.9966 ± 0.0004b	4,047.29 ± 722.25a	4,041.75 ± 783.16a
S	10.16 ± 0.42ab	0.9979 ± 0.0001a	4,313.95 ± 773.04a	4,290.89 ± 755.66a
M	10.13 ± 0.04ab	0.9967 ± 0.0001b	5,340.13 ± 278.26a	5,425.43 ± 404.23a
L	10.29 ± 0.15a	0.9980 ± 0.0004a	4,905.93 ± 1,166.02a	4,886.7 ± 1,137.19a

*Different letters indicate significant differences (p < 0.05) among canopy gap levels, based on a one-way ANOVA followed by an LSD test. “S” stands for small gaps; “M” stands for medium gaps; and “L” stands for large gaps*.

**Figure 3:**
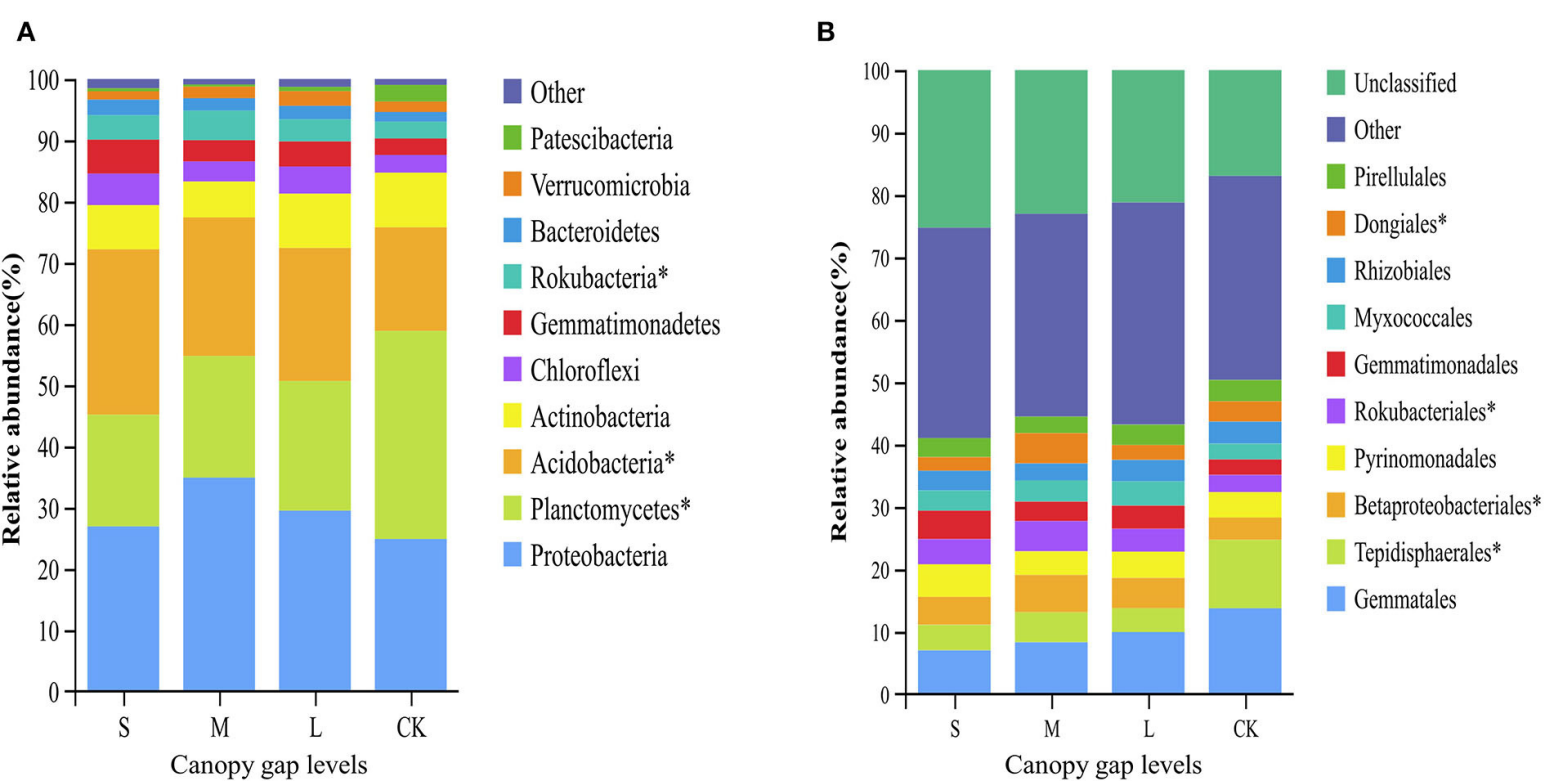
Relative abundance of bacteria taxa at the phyla level **(A)** and the orders level **(B)**. Only the bacteria in the top ten relative abundance are shown. A single asterisk indicates *p* < 0.05. “S” stands for small gaps; “M” stands for medium gaps; and “L” stands for large gaps.

**Figure 4:**
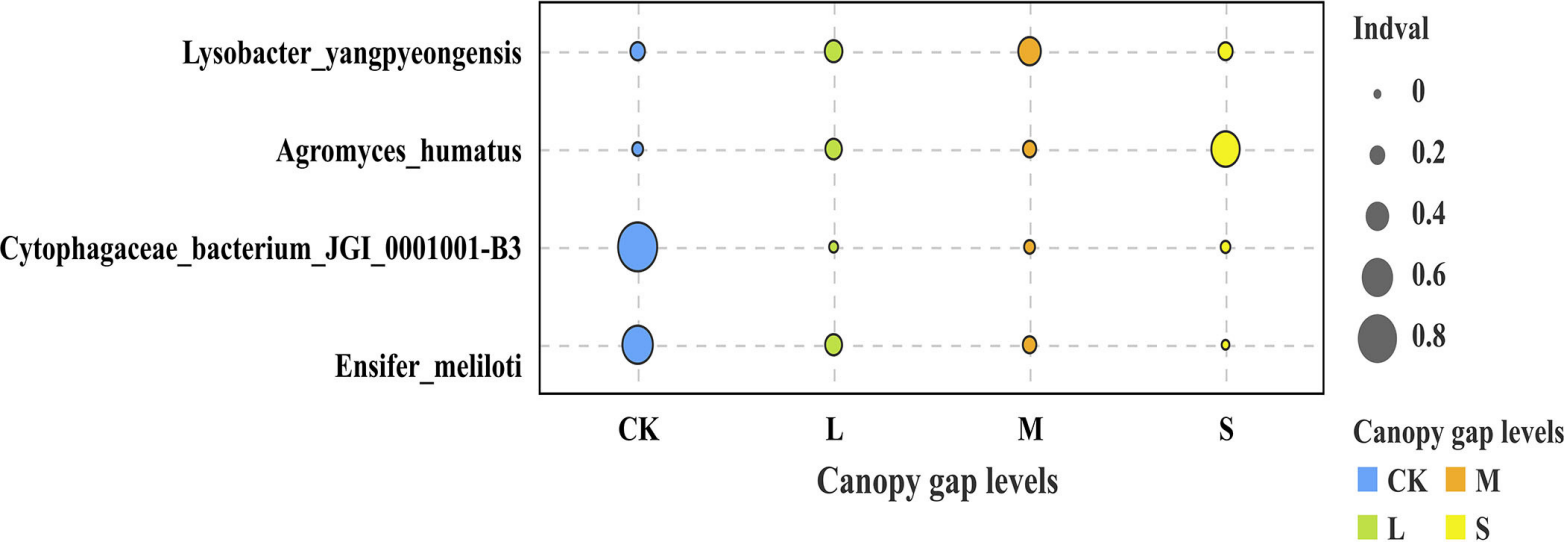
Soil bacteria community indicator species in the three different forest gaps. Only species with abundance value > 0, total proportion > 0.1%, and *p* < 0.05 are shown. “S” stands for small gaps; “M” stands for medium gaps; and “L” stands for large gaps.

Three bacterial phyla, namely, Proteobacteria, Planctomycetes, and Acidobacteria, accounted for 74.44% of the total sequences (Supplementary Table S2). The relative abundances of Planctomycetes, Acidobacteria, and Rokubacteria differed significantly across the plots with three different gap sizes at the phylum level (*p* < 0.05). The relative abundance of Planctomycetes in the CK plots (33.98 ± 11.82%) was significantly higher than that in the sampling plots with small (18.25 ± 0.85%), medium (19.85 ± 1.73%), and large (21.16 ± 4.11%) gaps. The relative abundance of Rokubacteria in the CK plots (2.78 ± 0.66%) was significantly lower than that of the plots with small (4.02 ± 1.56%) and medium gaps (4.85 ± 0.29%) (*p* < 0.05; Figure 3A). At the order level, the relative abundance of Tepidisphaerales in the plots with forest gaps was significantly lower than that of the CK plots (*p* < 0.05). However, the relative abundance of Betaproteobacteriales and Rokubacteriales was contrary to that of Tepidisphaerales. The relative abundance of Betaproteobacteriales and Rokubacteriales in the plots with forest gaps was significantly higher than that in the CK plots (*p* < 0.05), and their abundance was highest in the plots with medium gaps. The relative abundance of Dongiales in the plots with small gaps was significantly lower than in the CK plots but significantly higher than that in the plots with medium gaps (*p* < 0.05; Figure 3B). At the species level, the indicator values of *Lysobacter_yangpyeongensis, Agromyces_humatus, Cytophagaceae_bacterium_JGI_0001001-B3*, and *Ensifer_meliloti* were significantly different across the sampling plots with small, medium, and large gaps (*p* < 0.05; Figure 4). The functions of the top 10 bacteria with the highest relative abundance at level 2 were nearly similar in the sampling plots with different forest gap sizes (Supplementary Figure S2), in which a relative abundance greater than 10% was the main function, and included the metabolism of cofactors and vitamins, amino acid metabolism, and carbohydrate metabolism.

### Relationship between environmental variables and soil bacterial community

**Figure 5:**
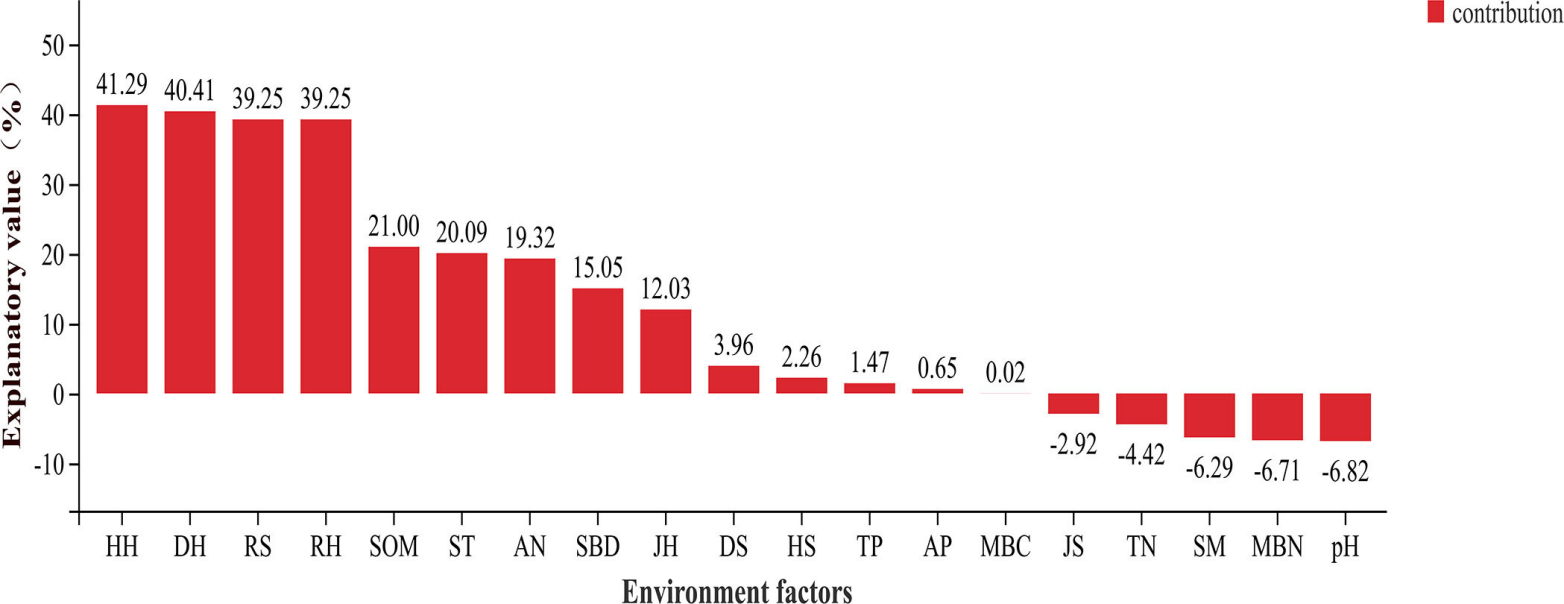
Variance partitioning analysis (VPA) of dominant phyla of soil bacteria and environmental factors.

**Figure 6:**
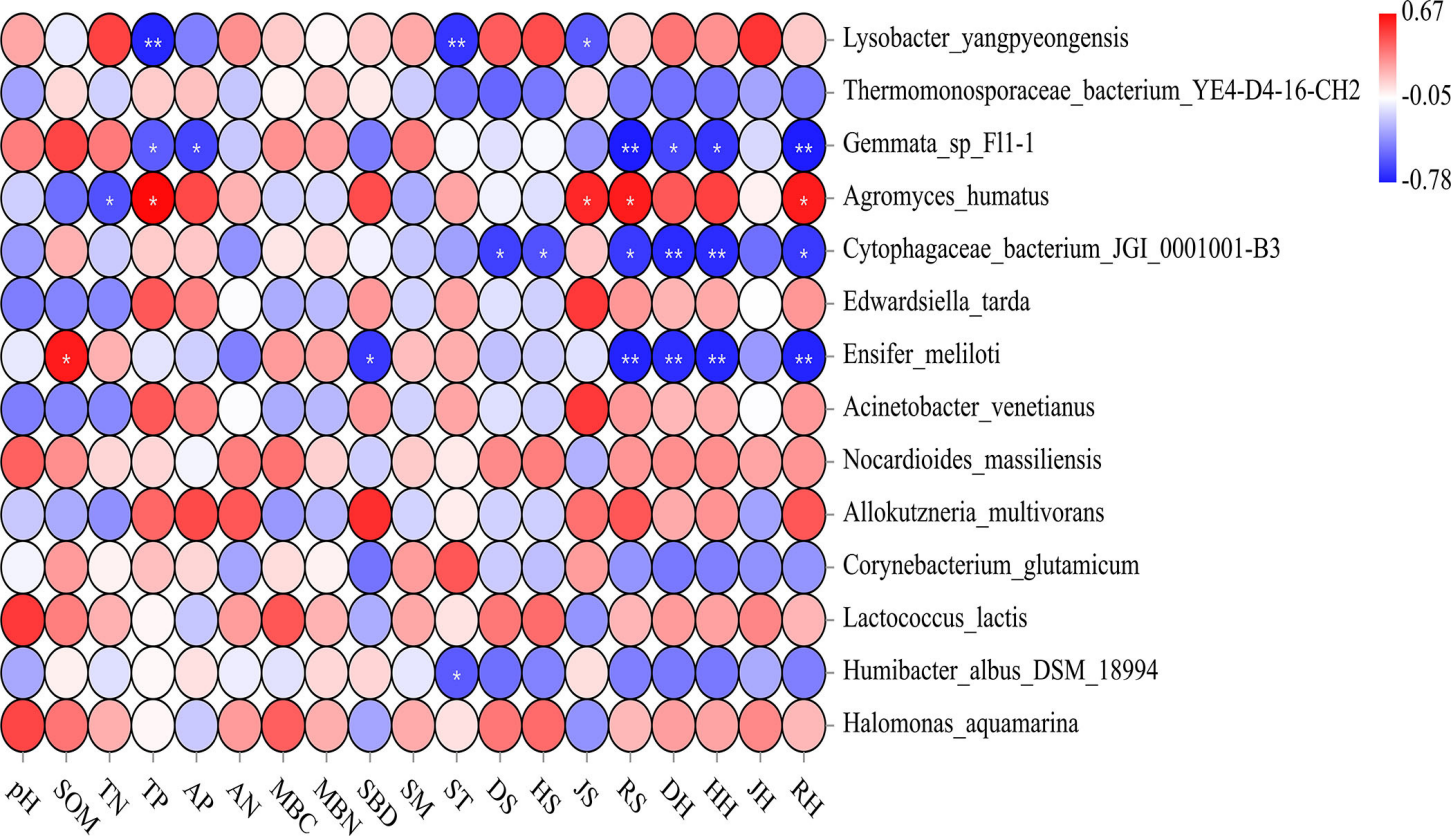
A heat map depicts the relative abundances of indicator species and Spearman's correlations between abundances of indicator species and environmental factors. Double asterisks indicate a *p-*value < 0.01; single asterisk indicates a *p-*value < 0.05. RH, DH, JH, and HH denote the richness index, the Simpson index, the Pielou index, and the Shannon index in the herb layer, respectively. RS, DS, JS, and HS denote the richness index, the Simpson index, the Pielou index, and the Shannon index in the shrub layer, respectively. This notation signifies the same meanings in the text. “S” stands for small gaps; “M” stands for medium gaps; and “L” stands for large gaps.

The Shannon and Simpson indices of the soil bacteria were mainly related to the understory plant diversity. Except for the Pielou index of the shrub and herb layers, the other plant diversity indices were positively correlated with the Shannon index of the soil bacteria (*p* < 0.05). The RS, DH, HH, and RH were positively correlated with the soil bacterial Simpson index (*p* < 0.05; Supplementary Table S3). The HH, DH, RS, and RH explained that the total variation in the composition of the soil bacterial community at the phylum level was much higher than the other environmental factors (Figure 5). The soil bacterial community was mostly related to understory plant diversity at the order level than the other properties of the soil (*p* < 0.05; Supplementary Figure S3). Similarly, the correlation between the indicator species of the soil bacterial community and understory plant diversity was higher than that between the indicator species and other properties of the soil. The relative abundance of *L. yangpyeongensis* was significantly negatively correlated with the JS, ST, and TP (*p* < 0.05), while that of *A. humatus* was significantly positively correlated with the RH, RS, JS, and TP (*p* < 0.05). The relative abundance of *C. bacterium_JGI_0001001-B3* was mainly affected by understory plant diversity and was significantly negatively correlated with the richness, Simpson, and Shannon indices (*p* < 0.05). The relative abundance of *E. meliloti* was significantly negatively correlated with the RH, HH, DH, and RS, but positively correlated with the SOM (*p* < 0.05; Figure 6).

**Figure 7:**
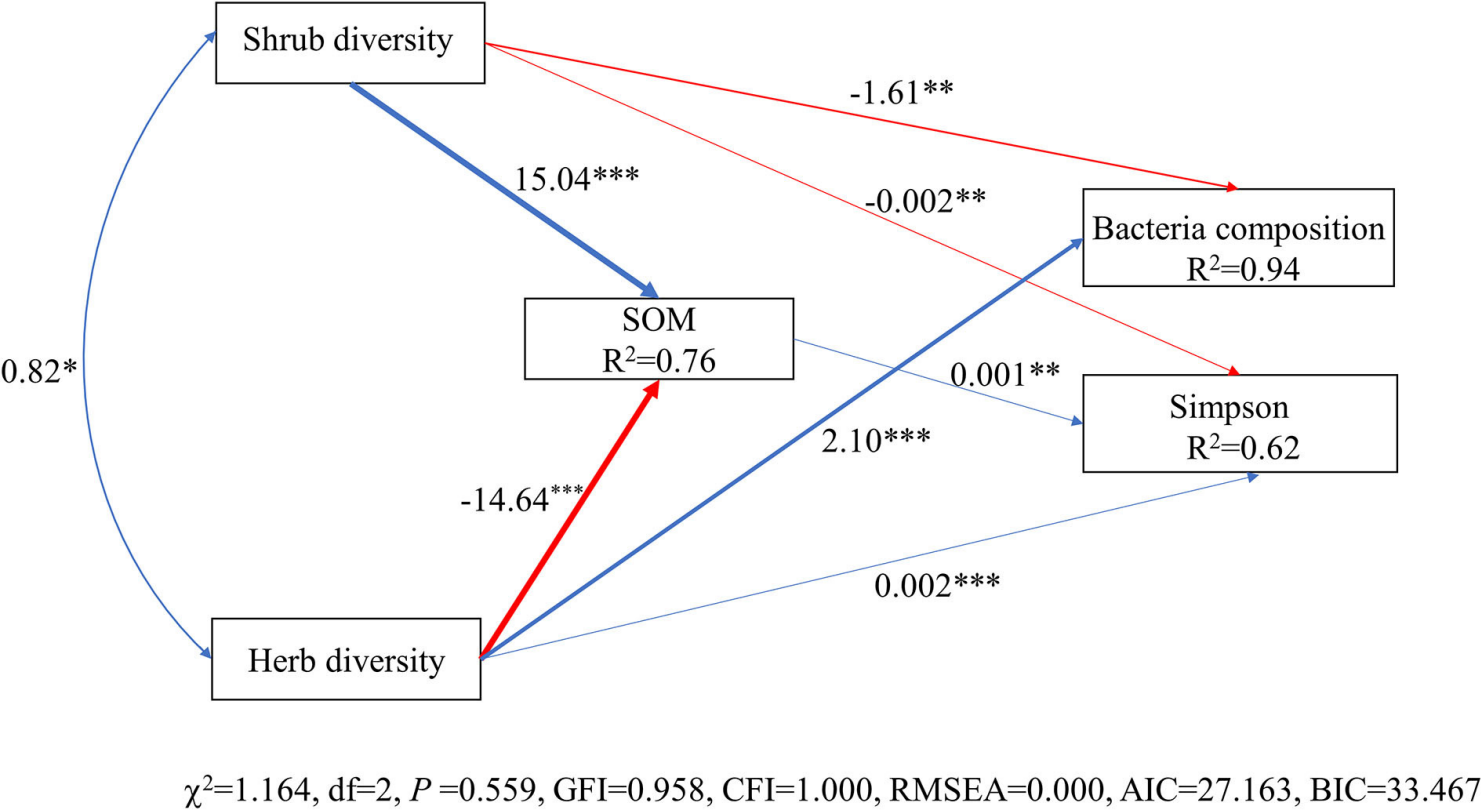
Structural equation model (SEM) of bacterial diversity and composition in relation to environmental factors. Solid arrows indicate significant effects (*P* < 0.05). Arrow width corresponds directly to the standardized path coefficient. The blue arrow represents the positive impact, and the red arrow represents the negative impact. *R*^2^ values associated with response variables indicate the proportion of explained variation by relationships with other variables. Values associated with arrows represent standardized path coefficients. Herb diversity: four plant diversity indices of the herb layer; SOM: soil organic matter; shrub diversity: four plant diversity indices of the shrub layer; bacterial composition: the sequencing data for bacteria at the phyla level; Simpson: Simpson index of bacteria community. ***P < 0.001; **P < 0.01; *P < 0.05.

**Figure 8:**
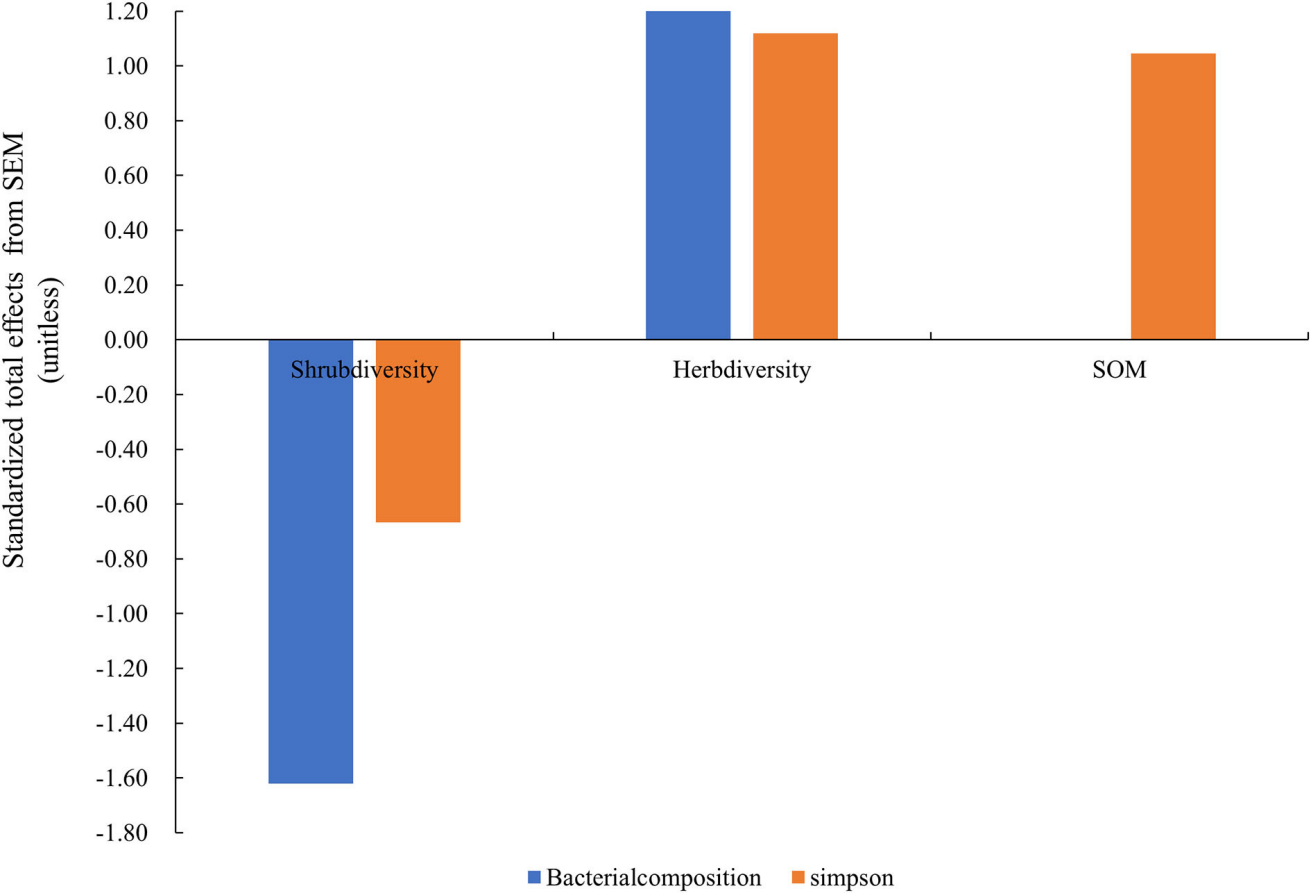
Standardized total effects (direct plus indirect effects) derived from the structural equation modeling. Herb diversity: four plant diversity indices of the herb layer; SOM: soil organic matter; shrub diversity: four plant diversity indices of the shrub layer; bacterial composition: the sequencing data for bacteria at the phyla level; Simpson: Simpson index of bacteria community.

The model constructed by SEM adequately fitted the data and standardized path coefficients, which indicated that the herb diversity and the shrub layer had a greater direct effect on the Simpson index and composition of the soil bacterial community than the SOM (Figure 7). Shrub diversity had a significant negative effect on the Simpson index and composition of the soil bacterial community (*P* < 0.01), while the diversity of the herb layer had a significant positive effect on the Simpson index and the composition of the soil bacterial community (*P* < 0.001). The SOM had a significant positive effect on the Simpson index through the regulation of understory plant diversity (*P* < 0.01). The shrub diversity and herb diversity accounted for 94% of the variation in the composition of the bacterial community and 62% of the variation in the Simpson index of the bacteria community. Notably, the direct effects of herb diversity on the composition and the Simpson index of the bacterial community were strongly positive, being 2.12 and 1.12, respectively, while the direct effects of shrub diversity on the composition and the Simpson index of the bacterial community were strongly negative, being −1.62 and −0.67, respectively. The SOM indirectly affected the Simpson index of the bacterial community by affecting the composition of the bacterial community (Figure 8).

Five principal component variables were extracted by PCA of 23 indices of understory plant diversity, bacterial community diversity, and physicochemical properties of the soil, and the cumulative contribution rates were as high as 88.06% (Supplementary Table S4). This indicated that the comprehensive indices of the first five principal components extracted with PCA represented most of the information of the 23 individual indicators. Compared to the CK plots, the plots with different forest gap sizes exhibited improved understory plant diversity, bacterial community diversity, and soil physicochemical properties, and the comprehensive evaluation values were in the order: L > S > M > CK (Table 3).

**Table 3:** Comprehensive appraisal value and sequencing of forest gaps.

**Canopy gap levels**	**Principal component**					**Comprehensive evaluation**	**Rank**
	***F*1**	***F*2**	***F*3**	***F*4**	***F*5**	***F***	
S	−0.62	1.47	0.22	1.00	0.30	0.26	2
M	0.66	−0.21	−1.31	−0.03	0.07	0.08	3
L	1.10	−0.13	0.92	−0.25	−0.26	0.50	1
CK	−1.15	−1.12	0.17	0.18	−0.11	−0.84	4

“*S” stands for small gaps; “M” stands for medium gaps; and “L” stands for large gaps*.

## Discussion

### Effects of forest gaps on soil properties and understory plant diversity

The formation of forest gaps increases rain flushing and solar radiation in the understory of forests and subsequently affects the temperature and physicochemical properties of the soil, in addition to altering the forest environment (Jianxin et al., 2016). However, the results of the present study demonstrated that forest gaps significantly affected only a minor aspect of the properties of soil. The loss of soil TN in plots with gaps is inconsistent with the results of this study compared to those obtained in studies on closed forests (Scharenbroch and Bockheim, 2007, 2008). The higher TN content in plots with medium and large gaps (Supplementary Table S1) is most likely to be attributed to the adequate light intensity that promotes seed germination and plant colonization, resulting in constant organic matter inputs, rhizodeposition, and nutrient release and recycling (Deng et al., 2018). The formation of forest gaps promotes the occurrence and succession of vegetation (Lu et al., 2018). In this study, we observed that the plant diversity in the herb layer was significantly more sensitive than that in the shrub layer (Table 1). In addition, the values of the four plant diversity indices of the herb layer in plots with forest gaps were significantly higher than those of the CK plots, which confirms our first hypothesis. The herb layer is more sensitive to environmental changes, is more resistant to stress, and can maintain the stability of the forest ecosystem. It plays a protective role, and an increase in the diversity of herbaceous plants indicates an improvement in plant community habitat (Von Oheimb and Härdtle, 2009; Catorci et al., 2013). The size of forest gaps directly or indirectly affects environmental heterogeneity, resource allocation, and vegetation renewal strategies, which consequently influence the species composition and community structure of the vegetation community (Diamond and Ross, 2016). Closed forests have a high canopy density and few light resources, which limit the growth and reproduction of several herbaceous plants (Mallik et al., 2014). Forest gaps are small-scale forest disturbances that increase photosynthetic active radiation in forests, prolong the light time, induce the gradual germination of certain plants in forest gaps that cannot be naturally renewed under the forest, and improve the structure of herbaceous plants (Keram et al., 2019). The Simpson, Shannon, and Pielou indices of the herb layer and the Shannon and richness indices of the shrub layer were highest in plots with large gaps compared to those of the plots with small and medium gaps. These differences could be attributed to the fact that plots with large gaps have higher light conditions, heterogeneity of canopy structure, and a wider living space for the plant community (Wu et al., 2022). Consequently, the understory vegetation receives abundant growth space, water, and heat, which is beneficial for nutrient and biomass accumulation by plants by making complete use of all kinds of resources (Márialigeti et al., 2016). The TN content is known to be the main limiting factor for plant growth and is closely related to the understory plant diversity (Lucasborja and Delgadobaquerizo, 2019; Lyu et al., 2021b), which was also observed in this study (Figure 1). We observed that the understory plant diversity was significantly affected by the available nutrients and TN content.

### Effects of forest gaps on the diversity and composition of the soil bacterial community

A previous study reported that soil bacteria are resilient or resistant to selective logging to some extent (Jin et al., 2019). The significant increase in the Shannon and Simpson indices of the soil bacterial community (Table 2) by the presence of large gaps could be related to the increased diversity of plant species which have well-developed root systems, and the intertwining of roots enhances the microbial activity and population in soils (Yang et al., 2015, 2018). As proposed in the second hypothesis, the bacterial communities in plots with different gap sizes were clearly differentiated by PCoA (Figure 2) and analysis of similarities (Supplementary Figure S1), which suggested that forest gaps of different sizes harbor different bacterial community structures.

Proteobacteria, Planctomycetes, and Acidobacteria were the dominant bacterial phyla in the weeping cypress forest (Supplementary Table S2). Some studies reported that these three soil bacterial phyla dominate bacterial communities in different geographical regions and soil types (Liu et al., 2013; Li et al., 2014), which agrees with the results of our study. Proteobacteria belongs to symbiotic or r-strategy: populations (Suh et al., 2014) and has an obvious niche competitive advantage in nutrient-rich environments. These bacteria participate in the carbon and nitrogen cycles during litter decomposition and are the dominant bacteria during litter decomposition (Vivanco et al., 2018). Some studies investigated the alterations in bacterial communities, and the common Acidobacteria are considered to be K-strategists (Zechmeister-Boltenstern et al., 2015; Yan et al., 2020). The results of this study demonstrated that the population of Acidobacteria increased significantly, while that of the Planctomycetes bacteria decreased significantly in plots with forest gaps (Figure 3A). Forest gaps alter the composition of bacterial communities *via* different life-history strategies. The bacterial communities in soils are involved in important biogeochemical cycles (Jenkins et al., 2017). Acidobacteria is a phylum of eosinophilic bacteria that grows well in acidic soils and is widely distributed in various environments (Shen et al., 2013; Liu et al., 2017). Therefore, Acidobacteria was found to be the dominant phylum in this study, even at pH values ranging between 7.80 and 8.10. Planctomycetes are ubiquitous, environmentally and biotechnologically important bacteria that are key players in global carbon and nitrogen cycles (Wiegand et al., 2018). Compared with the CK plots, several weeping cypress trees were removed from the canopy of plots with forest gaps, which reduced litter input. As Planctomycetes bacteria degrade various heteropoly saccharides and are involved in litter degradation (Ivanova et al., 2016), the relative abundance of Planctomycetes decreased significantly in plots with forest gaps. Kroeger et al. (2018) reported that carbon cycling is significantly altered by deforestation, which significantly increases the population of Rokubacteria in plots with forest gaps after deforestation. Previous studies indicated that Rokubacteria have diverse metabolic functions and metabolize a wide variety of carbon sources ranging from gaseous substrates to complex hydrocarbons. The vegetation alters the nutrient status of soils through root exudates and litter, providing different habitats for soil bacteria, which alters the functions of the soil bacterial community (Tolli and King, 2005). The indicator species can effectively predict the intensity of links between habitats and species and are therefore regarded as ecological indicators of environmental change and community type (Raiesi and Beheshti, 2015; Chai et al., 2019). Forest gaps improved soil nitrogen cycling, and significant differences were observed in the population of the nitrogen-fixing bacteria, *L. yangpyeongensis*, and *E. meliloti*, across the sampling plots with different gap sizes (Figure 4). The other two indicator species, *A. humatus* and *C. bacterium_JGI_0001001_B3*, have different life-history strategies, and further studies are necessary for understanding the roles of these indicator species in driving forest ecosystem succession in regions with artificial forest gaps. The primary function of metabolism involves the maintenance of bacterial survival *via* the ingestion of soil nutrients, including amino acids, energy, and carbohydrates (Jenkins et al., 2017). The vegetation absorbs nutrients for promoting the growth of the bacterial community through the activity of the main functional metabolic genes (Wu et al., 2016). Soil microorganisms include bacteria, fungi, and archaea, among others. In this study, the composition, diversity, and potential function of only the soil bacterial community were investigated. The structure of the soil microbial community requires further quantification in further studies.

### Understory plant diversity drives diversity and composition of soil bacterial community

The composition of soil bacterial communities is strongly influenced by the above-ground components of ecosystems (Steinauer et al., 2015; Vitali et al., 2016). The above-ground plant community is closely related to the underground microbial community, forms a systematic entity that maintains the function of the ecosystem, and can strongly affect the structure of the soil microbial community through the comprehensive influence of nutrient availability and the physicochemical state of the soil (Chu et al., 2011; Wang et al., 2015). We observed that the dominant soil bacterial community and bacterial diversity in the cypress forests were mainly driven by understory plant diversity (Supplementary Table S3; Figure 5; Supplementary Figure S3), which confirmed our third hypothesis. One explanation for this observation is niche complementarity, which postulates that higher forest functioning is attributed to the higher species composition and diversity of functional traits, resulting from the efficient utilization of resources by species coexisting within a community (Tilman, 1999). The higher plant diversity provides a more diversified litter composition resulting from the increased diversity of plant biochemical components, which provides microorganisms with a more diverse nutrient pool and increases bacterial diversity (Eisenhauer et al., 2010). According to the gap partitioning hypothesis, gaps alter the physical structure of forest stands and create a gradient of resource conditions in forest understories (Denslow, 1987). In addition, the felled trees and artificial trampling of plants in the forest during the processes of transportation and migration for the construction of forest gaps increase plant residue inputs, thus providing more substrates for soil microorganisms, which results in a more active and abundant microbial community (Eisenhauer et al., 2010). Second, the indicator species in the soil bacterial community in the forest gaps were closely related to the understory plant diversity (Figure 6). This could be attributed to the fact that increased plant diversity increases carbon input into the rhizosphere of the microbial community (Lange et al., 2015). Plant roots must compete with the roots of other plants for space, water, and nutrients. In addition, plants produce various chemical compounds that attract beneficial microbes (Philippot et al., 2013; Meng et al., 2019), thus altering the microbial activity and influencing the indicator species.

The relationship between soil environmental variables and soil bacterial community structure was quantified with SEM, which indicated that the accumulation of SOM and the increased understory plant diversity significantly promoted the development of the soil bacterial community (Figures 7, 8). Plant–microbe–soil interactions play a key role in determining nutrient cycling. The understory plant diversity promotes the diversity and composition of soil microbes by providing diversified food resources, including carbon, nitrogen, and organic matter, for soil microorganisms, which promotes the absorption, utilization, decomposition, and mineralization functions of soil microorganisms (Klironomos et al., 2011). Generally speaking, a higher plant diversity usually promotes plant productivity. Therefore, plant produces more carbon sources for soil microbial communities. As plant species differ with respect to the quantity and quality of resources returned to the soil, the presence of different plant species may have an important impact on the composition and diversity of soil microflora (Wardle et al., 2004). Therefore, the increased understory plants significantly affected the composition and Simpson index of the bacterial communities. Numerous studies reported that changes in the above-ground plant diversity induce changes in the metabolic activity, composition, and diversity of underground bacterial communities (Grüter et al., 2006; Zhang et al., 2017), which was consistent with the results of our study. The understory vegetation promotes the decomposition and transformation rate of litter and therefore returns higher quantities of nutrients to the soil. Soil bacteria are more likely to use easily decomposed organic matter in the soil and are competitive in the utilization of soil nutrients (Guo et al., 2021). Therefore, the SOM indirectly affected the alterations in the structure of the soil bacterial community. However, the alterations in the composition and diversity of bacterial communities could not be fully explained by the diversity of understory plants alone. The factors affecting the changes in the soil bacterial community may also include certain unmeasurable characteristics, including the carbon chemistry of the litter, changes in soil enzymatic activities, or plant functional traits, which require further investigation.

### Effects of forest gaps on forest management

Forest gaps are one of the common disturbance modes in forest ecosystems, which can drive forest regeneration and succession (Splechtna et al., 2005). The results of the comprehensive evaluation of understory plant diversity, bacterial diversity, and environmental factors were in the order: L > S > M > CK (Table 3). This indicated that the formation of forest gaps of different sizes in the same climate induces the formation of different microclimatic environments (Zhu et al., 2021). These differences can alter the structure and diversity of bacterial communities to a certain extent, which subsequently affects the material and energy cycles in forest ecosystems (Yang et al., 2018). In the early stage of forest gap community succession, the improvement in the soil environment and the increase in plant diversity were most pronounced in plots with large gaps. This could be attributed to the fact that there was a marked difference between the environmental heterogeneity of plots with large gaps with those of plots with small and medium gaps. Environmental heterogeneity in turn promotes forest regeneration (Uriarte et al., 2018) and alters the abundance and distribution of biotic and abiotic resources (Denslow, 1987), which increases biodiversity by maintaining the gap between microenvironment and forest microclimate. The comprehensive effect of small forest gaps on soil bacterial diversity, plant diversity, and soil properties was greater than that of the medium forest gaps. A plausible reason could be that the soil environment of small gaps responds more quickly to interference in the forest than medium gaps, and small gaps are more conducive to the accumulation of soil phosphorus and increased plant diversity in the herb layer. As plants directly use inorganic phosphorus, phosphorus is a limiting factor for plant growth (Lyu et al., 2021b), which requires the hydrolysis of organophosphorus compounds by bacterial and fungal phosphatases prior to absorption by plants (George et al., 2002). Muscolo et al. (2007) reported that phosphatase activity gradually decreases in plots with medium or large gaps, which is consistent with the reduction in the phosphorus content. Herbaceous vegetation is regarded as the key plant functional group that affects soil microbial communities (Milcu et al., 2008). The characteristics of soils in plots with small gaps provide a more favorable environment for the growth of microorganisms and herbaceous vegetation and promote the functions of soil microorganisms and forests (Wang et al., 2021). In summary, the use of near-natural management practices with planted forests has become an important concept in ecosystem management. The creation of forest gaps is regarded as an indispensable forest management measure for improving the above-ground and underground ecosystems of forest plantations. The formation of forest gaps may have practical value in guiding forest management plans. The results of this study demonstrated that large gaps are more beneficial to improving the quality of forest plantations in the initial stages of near-natural management strategies for forest plantations. However, it is expected that the strategy will be disrupted over years of community succession, which requires further long-term monitoring studies.

## Conclusion

Overall, this study provided insights into the reasons underlying the changes in bacterial community structure and composition induced by the construction of forest gaps in weeping cypress plantations. The construction of forest gaps significantly increased the diversity of the herb layer and altered the composition of the soil bacterial community. Large gaps significantly increased the Shannon and Simpson indices of soil bacterial diversity. The understory plant diversity significantly affected the composition and diversity of the soil bacterial community compared to the other soil factors. The results of the present study demonstrated that large gaps had the greatest effect on understory plant diversity, which affected the soil bacterial community in the early stage of community succession and could be applied to increasing the ecological benefit of near-natural forest management strategies for forest plantations. The present study primarily focused on obtaining insights into the bacterial community of soils, and insights into the soil fungal community are necessary for subsequent research studies. The study needs to be performed on a long-term scale for obtaining better insights into the effects of soil microbial communities on forest ecosystems.

## Data availability statement

The datasets presented in this study can be found in online repositories. The names of the repository/repositories and accession number(s) can be found below: https://www.ncbi.nlm.nih.gov/, BioProject: PRJNA784027.

## Author contributions

QL, YL, and XianwL conceived the study and designed the methodology. QL, YL, SL, YZ, and XiangL conducted field sampling. QL and YL performed the laboratory work. QL analyzed the data and wrote the first draft of the manuscript. GH, GC, and KZ assisted with revising the draft manuscript. CF provided laboratory resources. All authors approved the final manuscript.

## Funding

This study was supported by the Forest Ecosystem Improvement in the Upper Reaches of Yangtze River Basin Program of the World Bank (2019-510000-02-01-400761), the German Government loans for Sichuan Forestry Sustainable Management (Grant No. G1403083), and the Key Sci-tech Project of the 12th 5-year Plan of China (Grant No. 2011BAC09B05).

## Conflict of interest

The authors declare that the research was conducted in the absence of any commercial or financial relationships that could be construed as a potential conflict of interest.

## Publisher's note

All claims expressed in this article are solely those of the authors and do not necessarily represent those of their affiliated organizations, or those of the publisher, the editors and the reviewers. Any product that may be evaluated in this article, or claim that may be made by its manufacturer, is not guaranteed or endorsed by the publisher.
