# Supplementary material for: Forest gaps alter the soil bacterial community of weeping cypress plantations by modulating the understory plant diversity
Source: Front Plant Sci. 2022 Aug 19;13:920905. doi: 10.3389/fpls.2022.920905 (PMC9437579; doi:10.3389/fpls.2022.920905)
Supplement: Supplementary file 1 [file Data_Sheet_1.docx]

Supplementary Material

# Supplementary Tables

**Supplementary Table S1** Soil properties among different canopy gap levels.

| Canopy gap levels | pH | SOM (g/kg) | TN (g/kg) | TP (g/kg) | AP (mg/kg) | AN (mg/kg) | MBC (g/kg) | MBN (g/kg) | SBD (g/cm³) | SM (%) | ST (℃) |
| --- | --- | --- | --- | --- | --- | --- | --- | --- | --- | --- | --- |
| CK | 7.96±0.17a | 24.32±4.14ab | 1.02±0.08b | 0.55±0.01b | 6.33±0.58b | 59.67±2.17a | 264.96±31.78a | 1.16±0.24ab | 1.18±0.25b | 22.62±4.64a | 20.63±0.29a |
| S | 7.87±0.09a | 9.74±2.15c | 0.87±0.02c | 0.63±0.00a | 8.15±0.41a | 75.47±7.19a | 168.06±4.39a | 0.56±0.31b | 1.49±0.07a | 21.08±0.50a | 20.63±0.58a |
| M | 8.03±0.16a | 15.35±7.02bc | 1.18±0.08a | 0.45±0.03c | 5.54±1.01b | 71.87±17.97a | 267.55±112.32a | 0.58±0.08b | 1.26±0.09ab | 24.99±5.98a | 20.40±0.00a |
| L | 8.07±0.13a | 25.07±4.66a | 1.20±0.10a | 0.52±0.01b | 5.44±1.13b | 74.51±3.40a | 270.75±83.51a | 2.18±1.08a | 1.13±0.09b | 25.84±1.32a | 20.67±0.12a |

Data are expressed as the average ± SD. Different letters in the same column denote statistically significant at *p* <0.05, based on a one-way ANOVA followed by an LSD test.

**Supplementary Table S2** Composition of soil bacterial community at phyla level. Only the bacteria in the top ten relative abundance are shown.

| Phylum | Relative abundance % (mean abundance) |
| --- | --- |
| Proteobacteria | 29.06 |
| Planctomycetes | 23.31 |
| Acidobacteria | 22.07 |
| Actinobacteria | 7.71 |
| Chloroflexi | 3.94 |
| Gemmatimonadetes | 3.94 |
| Rokubacteria | 3.81 |
| Bacteroidetes | 2.08 |
| Verrucomicrobia | 1.83 |
| Patescibacteria | 1.07 |

**Supplementary Table S3** Pearson’s correlations of understory plant diversity, soil properties and soil bacterial diversity (* *p* < 0.05, ** *p* < 0.01).

| Indices | Shannon | Simpson | Chao1 | ACE |
| --- | --- | --- | --- | --- |
| SOM | -0.0485 | -0.042 | 0.020 | 0.010 |
| TN | 0.244 | -0.112 | 0.488 | 0.497 |
| TP | -0.11 | 0.465 | -0.527 | -0.538 |
| AP | -0.107 | 0.23 | -0.394 | -0.401 |
| AN | 0.496 | 0.396 | 0.107 | 0.070 |
| MBC | -0.036 | -0.109 | 0.139 | 0.184 |
| MBN | 0.354 | 0.347 | 0.278 | 0.256 |
| SBD | 0.113 | -0.001 | 0.038 | 0.027 |
| SM | 0.159 | 0.086 | 0.180 | 0.182 |
| ST | 0.121 | 0.442 | -0.029 | -0.039 |
| pH | 0.407 | 0.124 | 0.389 | 0.416 |
| DS | 0.623* | 0.389 | 0.542 | 0.547 |
| HS | 0.579* | 0.333 | 0.535 | 0.538 |
| JS | -0.211 | 0.149 | -0.431 | -0.440 |
| RS | 0.671* | 0.717** | 0.224 | 0.212 |
| DH | 0.698* | 0.603* | 0.397 | 0.398 |
| HH | 0.705* | 0.656* | 0.347 | 0.344 |
| JH | 0.509 | 0.249 | 0.525 | 0.548 |
| RH | 0.519 | 0.401 | 0.510 | 0.506 |

RH, DH, JH, and HH denote the Richness index, Simpson index, Pielou index, and Shannon index in the herb layer, respectively. RS, DS, JS, and HS denote the Richness index, Simpson index, Pielou index, and Shannon index in the shrub layer, respectively.

**Supplementary Table S4** The principal component (PC) eigenvalues and rate of variance explained.

| Index | Principal component 1 | Principal component 2 | Principal component 3 | Principal component 4 | Principal component 5 |
| --- | --- | --- | --- | --- | --- |
| Eigenvalue | 8.68 | 6.63 | 2.16 | 1.66 | 1.13 |
| Explained/ % | 37.75 | 28.83 | 9.37 | 7.20 | 4.90 |
| Cumulative variance explained/ % | 37.75 | 66.59 | 75.96 | 83.16 | 88.06 |

# Supplementary Figures


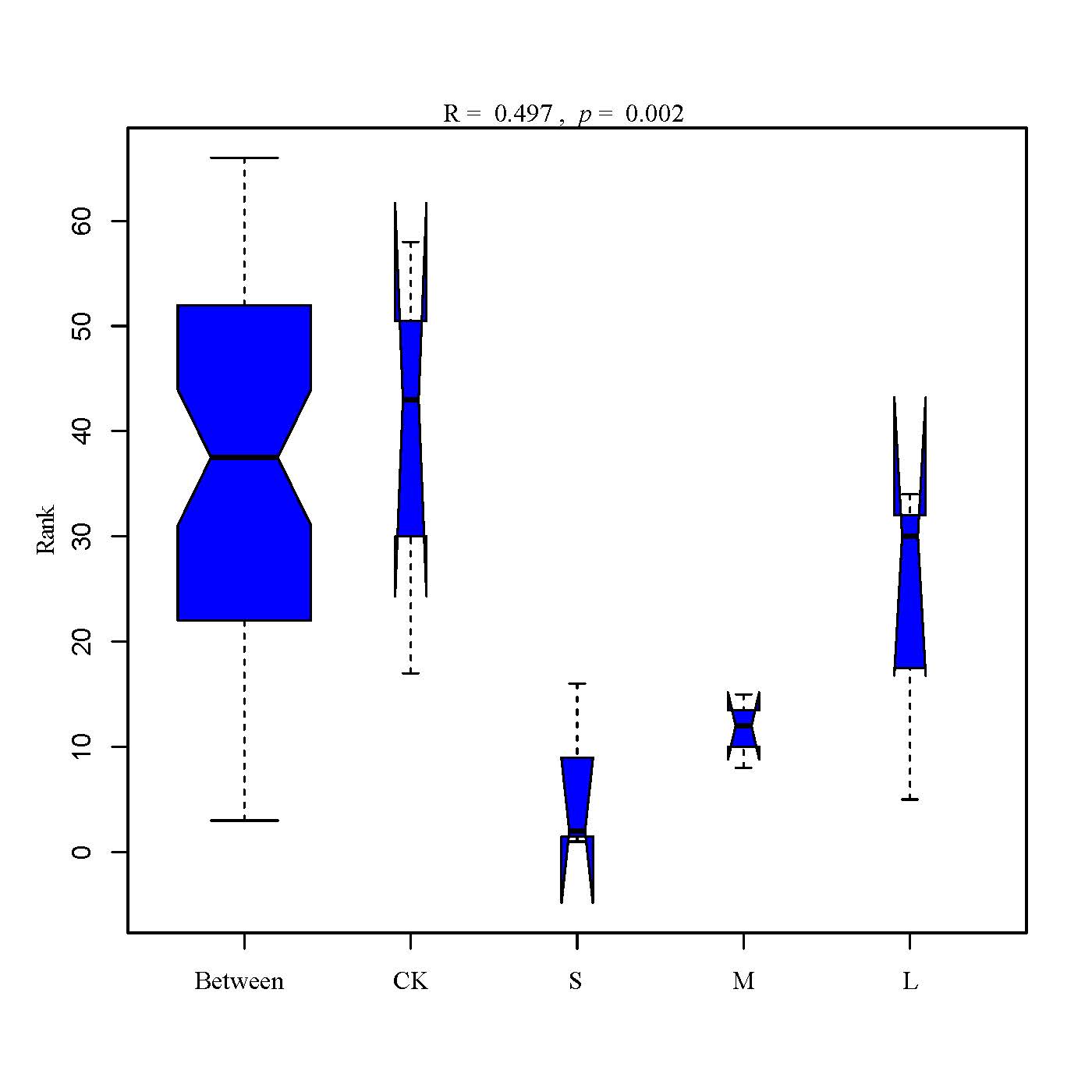


**Supplementary Figure 1.** Analysis of similarities of the soil bacterial community.

“S” stands for small gaps; “M” stands for medium gaps; “L” stands for large gaps.


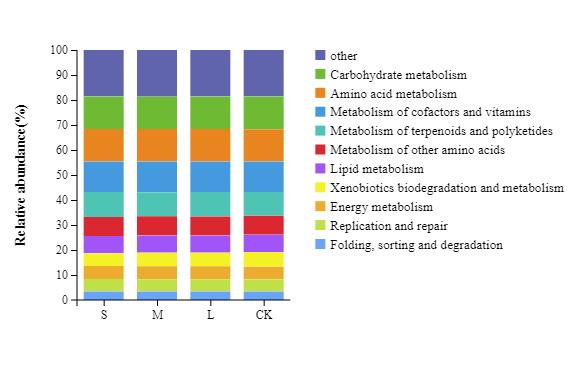


**Supplementary Figure 2.** The relative abundances of the function of soil bacterial community.

“S” stands for small gaps; “M” stands for medium gaps; “L” stands for large gaps.


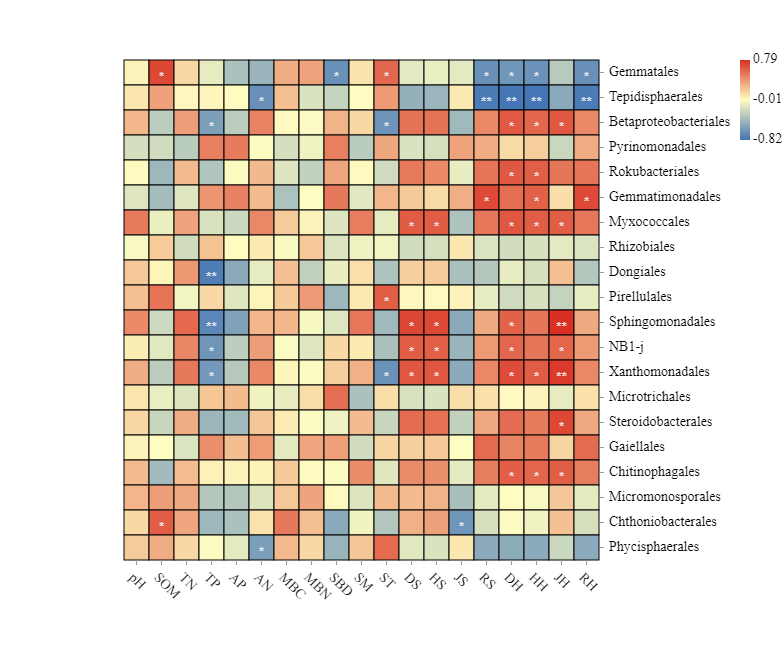


**Supplementary Figure 3.** Spearman’s correlations between abundances of bacteria orders and environmental factors. Double asterisks indicate *p* < 0.01; single asterisk indicates *p* < 0.05. “S” stands for small gaps; “M” stands for medium gaps; “L” stands for large gaps.
